# Plasma oxidized low density lipoprotein cholesterol correlates inversely with testosterone in young adult male smokers

**DOI:** 10.11604/pamj.2014.19.241.3354

**Published:** 2014-11-04

**Authors:** Maria Onomhaguan Ebesunun, Olurakinyo Lanre Bankole, Olayiwola Oduwole

**Affiliations:** 1 Chemical Pathology& Immunology, Obafemi Awolowo College of Health Sciences Olabisi Onabanjo,University, Sagamu Campus, Nigeria; 2 Institute of Reproductive and Developmental Biology, Imperial College London, Faculty of Medicine, Hammersmith Hospital Campus, London W12 0NN

**Keywords:** Cigarette, CVD, Ox-LDLC, testosterone, cholesterol

## Abstract

**Introduction:**

There are indications that oxidized low density lipoprotein cholesterol (Ox-LDLC) may play an important role in cardiovascular disease (CVD) events. In most developing countries, the interplay between the different lipid fractions and cigarette smoking has not been studied. This study assessed the effect of cigarette smoking on the alterations in plasma lipid fractions and their associations with the gonadal hormone, testosterone (T).

**Methods:**

One hundred and sixty male participants, consisting of eighty smokers and eighty apparently healthy non-smokers were recruited. Anthropometric indices and biochemical parameters were determined using standard procedures.

**Results:**

Significant increases were obtained in plasma total cholesterol (TC), triglyceride (TG), oxidized low density lipoprotein (Ox-LDLC) and Ox-LDLC/TT ratio (p<0.001) in smokers compared with the non-smokers. Plasma high density cholesterol (HDLC) (p<0.001) was significantly reduced in smokers compared with the non-smokers. The plasma mean T result was not significantly different from the non-smokers, but inversely correlated with Ox-LDLC and significantly correlated with the lipids and lipoproteins. Significantly high plasma TC, TG and LDLC (p<0.001) and low HDLC (p<0.001) were also obtained in smokers when co-founding factors such as duration and number of cigarette smoked per day were applied.

**Conclusion:**

This study showed an inverse correlation between Ox-LDLC and testosterone as well as strong association between the number of tobacco and cigarettes usage per day. These changes in part, could be major causes of premature CVD and decreased fertility in young adults.

## Introduction

The health consequences of smoking cigarettes and other tobacco related products are well known. They are important major health hazard in the development of atherosclerosis and cardiovascular disease (CVD) [1]. The connections between cigarette smoking and CVD have been addressed by several investigators [2–4]. In the INTERHEART study, Frick et al [5] indicated an overall population attributable risk of about 38% between smoking and myocardial infarction. Tobacco smoke in general contains numerous compounds majorly among which are nicotine, carbon monoxide and various carcinogens [3]. These compounds do have effects on the metabolic and biological processes of the body including the modification of lipoproteins, especially the LDLC [6], the oxidized form of which has been shown to accumulate excessively in arterial walls, leading to increased peroxidation both in vitro and in vivo [7]. Recent experimental and clinical data also suggest that exposure to cigarette and other tobacco related smoke increases oxidative stress, thereby compounding the potential mechanism(s) for initiating cardiovascular dysfunction through the activities of nicotine in cigarettes which enhances fat oxidation [8]. Experimental and clinical data [9] indicates that nicotine in cigarette smoke changes lipoproteins, particularly LDLC into the oxidized form(ox-LDLC) thus quickening its uptake by macrophages, thereby leading to the progression of atherosclerosis [3]. There is also a growing interest following several studies on the link between cigarette smoking and low testosterone levels in men in relation to the development of coronary heart disease[10, 11]. Overall, males have been shown to have a higher risk of CVD in comparison to premenopausal women, with the incidence in women increasing after menopause and a concomitant decrease in the gender difference in mortality and morbidity [12, 13]. In a developing country like Nigeria, where there is not much enlightenment on the implications and adverse effects of smoking cigarette and other tobacco related products are not known, a sizeable number of the male population are involved in this habit. The aim of this study therefore, was to ascertain the relationship between smoking, the different fractions of lipids and gonadal hormone. The results we believe will highlight the relationship between these parameters in our targeted subjects.

## Methods

One hundred and sixty apparently healthy male participants consisting of eighty smokers (44.4±1.7 years) and eighty non-smokers (35.1±1.0 years) were recruited for this study. The smokers were recruited from motor parks and military barracks; areas where the habit is a common occurrence. The smokers consumed at least 4 sticks of cigarette daily. Demographic data such as lifestyle pattern, smoking habit, alcohol use, socioeconomic status and medical history were obtained from each participant based on questionnaire adapted from the work of Ebesunun et al, [14]. The controls were men who had never been involved in cigarette smoking at any stage of their life. Informed consent was also obtained from participants before commencement of study.

### Anthropometric measurements

The weight of each participant in kilogram was taken using a Seca adult weighing scale. The height in centimeter was taken using a fixed meter rule without foot wear. The body mass index (BMI) in kg/m^2^ of each subject was calculated using weight/height^2^

### Sample collection

Blood samples were collected from all subjects after an overnight fast (10-12 hours) into EDTA bottles. The blood samples were immediately placed on ice bag and plasma was separated within short time of collection. The plasma samples were thereafter stored in at -20^°^ C until analyzed for biochemical parameters.

### Biochemical analysis

Total cholesterol was determined using the method of Alain et al, [15] while the triglyceride was estimated by the enzymatic method of David and Buccolo [16]. LDLC and VLDLC were precipitated out by the addition of phosphotungstic acid in the presence of magnesium ions. After centrifugation, the cholesterol concentration in the supernatant representing the HDLC fraction was determined with the method used for total cholesterol estimation. The LDLC was calculated using the formula of Friedwald et al, [17]. Ox-LDLC was analyzd using an Enzyme Immunoaborbent Assay (EIA) method based on the principle of competitive binding between Ox-LDLC in the test sample and oxidized LDL-HRP conjugate for a constant amount of rabbit anti-oxidized LDLC. Testosterone analysis was based on the principle of competitive binding between T in the test sample and testosterone-HRP conjugate for a constant amount of rabbit anti-testosterone. Accuracy and precision of biochemical tests were monitored by the inclusion of commercial quality control samples with each batch of assay.

### Statistical analysis

All results were subjected to statistical analysis using SPSS version 14 software (SPSS Inc. Chicago, Illinois) for windows. Student t-test was used for comparison of variables. Post Hoc analysis was also carried out. Pearson correlation coefficient was used to assess the relationship between variables. The results were expressed in mean ± S.E and the level of significance was accepted at p < 0.05.

## Results

Results are presented in tables 1-3 below: Table 1 shows biophysical and biochemical parameters in smokers and non-smokers. Smokers were older than non-smokers (p<0.001). There were significant increases in the plasma TC, TG, LDLC, Ox-LDLC and Ox-LDLC/TT ratio (p<0.001) in smokers when compared with the corresponding non-smokers. Plasma HDLC was significantly reduced in smokers compared with the corresponding non-smokers (p<0.001). There were no significant changes in BMI, total testosterone (TT) and LDLC/Ox-LDLC ratio in both smokers and non-smokers. 

**Table 1:** Biophysical and biochemical parameters of smokers and non-smokers(Mean + S.E.M)

Variables	Smokers n=80	Non-smokers n=80	t-value	p-value
BMI (kg/m^2^)	21.3±3.6	22.4±2.8	.86	NS
TC(mg/dl)	211.7±1.3	174.4±2.6	-12.7	p<0.001
TG (mg/d)	129.9±1.2	115.2±2.4	-5.6	p<0.001
HDLC(mg/dl)	40.2±0.8	47.1±0.7	6.8	p<0.001
LDLC(mg/dl)	143.7±1.8	102.6±2.8	-12.3	p<0.001
Ox-LDL (ng/dl)	65.4±0.7	44.4±0.4	-19.8	p<0.001
TT (ng/dl)	4.7±0.1.	4.9±0.1	.73	NS
LDLC/Ox-LDLC	13.9±0.1	9.1±0.1	.106	NS
Ox-LDLC/TT	13.9±0.1	9.1±0.1	27.1	p<0.001
Age (years)	44.4±1.7	35.1±1.0	-11.2	p<0.001

BMI=Body Mass Index. TC=Total Cholesterol. TG=Triglyceride.

TT =TotalTestosterone HDLC=High Density Lipoprotein Cholesterol.

LDLC= Low DensityLipoprotein Cholesterol. Ox-LDLC=Oxidized LDLC.

p= Level of significance. NS= not significant.

Table 2 shows the biophysical and biochemical parameters in smokers based on the duration (years) of cigarettes smoked. These were made up of two groups; group 1 consisted of smokers that have been smoking between 5 to 13 years, while group 2 consisted of those that have smoked between 14 to 22 years. Smokers in group 1 were young than smokers in group2 (p<0.001). The BMI of the two groups were not significantly different. The plasma TC, TG, LDLC, Ox-LDLC and Ox-LDLC/TT ratio of smokers in group 2 were significantly higher (p<0.001) when compared with the corresponding values in group 1. The HDLC was significantly lower (p<0.001) in group 2 smokers than the corresponding group 1 smokers. Plasma TT and LDLC/Ox-LDLC ratio (p>0.001) were not significantly different in the two groups. Comparison of parameters between military men and motor park vendors did not show any significant changes between the groups.

**Table 2:** Biophysical and biochemical parameters of smokers based on the duration (in years) of cigarette smoked. (Mean+S.E.M)

Variables	GP1=5-13 years(n=35)	GP2=14-22 years (n=45)	t- value	p-value
BMI (kg/m^2^)	22.3±3.6	21.7±2.8	10.2	NS
TC (mg/dl)	206.4±1.5	214.3±2.6	-9.7	p<0.001
TG (mg/dl)	126.1±1.2	132.8±2.4	-8.6	p<0.001
HDLC (mg/dl)	39.8±0.8	36.4±0.7	11.8	p<0.001
LDLC (mg/dl)	132.7±1.8	157.2±2.8	-18.3	p<0.001
Ox-LDLC(ng/dl)	55.6±0.7	67.4±0.4	-19.8	p<0.001
TT (ng/dl)	4.5±0.1	4.7±0.1	5.3	NS
LDLC/Ox-LDLC	2.4±0.1	2.3±0.1	9.6	NS
Ox-LDLC/TT	12.9±0.1	14.3±0.1	-24.1	p<0.001
Age (years)	32.8±1.7	39.1±1.0	-16.8	p<0.001

BMI=Body Mass Index. TC=Total Cholesterol. TG=Triglyceride.

TT =TotalTestosterone HDLC=High Density Lipoprotein Cholesterol.

LDLC= Low DensityLipoprotein Cholesterol. Ox-LDLC=Oxidized LDLC.

GP= group.p = Level of significance. NS= not significant.

**Figure 1:**
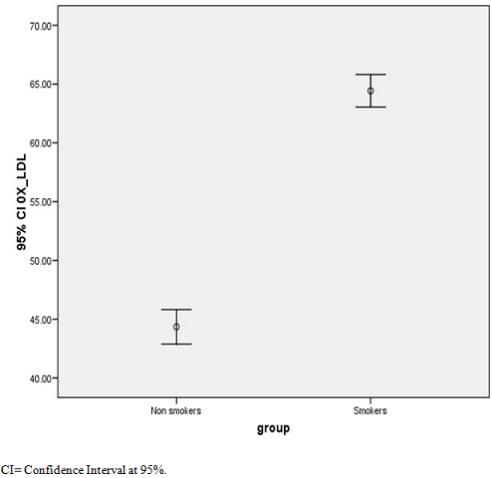
Oxidised-low density lipoprotein cholesterol in smokers and non smokers

**Figure 2:**
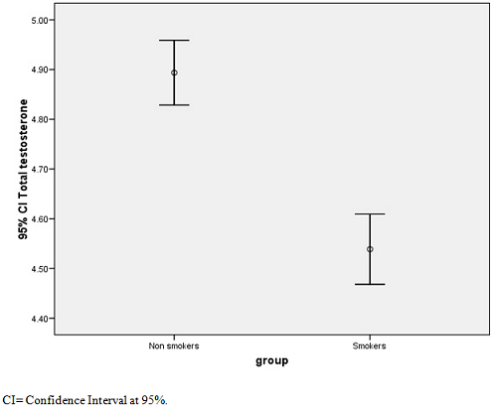
Total testosterone in smokers and non smokers

Table 3 shows Pearson correlation (r) coefficient, this indicated that plasma TC was significantly correlated with HDLC (r=0.958,p<0.01), LDLC(r=0.989, p<0.01) ox-LDLC(r=0.974.p<0/01) and TT(r=0.632,p<0.05).HDLC was inversely correlated with LDLC(r= -0.975,p<0.01) and Ox-LDLC(r= 0.941,P<0.01).Ox-LDLC was inversely correlated with TT (r=-0.649, p<0.05). Figure 1 shows a graph of Ox-LDLC in smokers and non-smokers. The smokers showed significantly higher value of plasma Ox-LDLC. Figure 2 shows a graph of testosterone in smokers and non-smokers. The smoker had a lower mean plasma testosterone.

**Table 3:** Pearson correlation coefficeint of biochemical parameters in smokers

Variables	TC (mg/dl)	HDLC (mg/dl)	LDLC(mg/dl)	Ox-LDLC(ng/dl)	TT (ng/dl)
TC (mg/dl)		.958**	.989**	.974**	.632*
HDLC (mg/dl)	958**		-.975**	.955**	.577*
LDLC (mg/dl)	.989**	-.957**		.981**	.615*
Ox-LDLC (ng/dl)	.974	.955**	.981**		-.649*
TT (ng/dl)	.632*	.577*	.615*	-.649*	

Pearson Correlation coefficient significant at

** p<0.01

* P<0.05

TT =TotalTestosterone HDLC=High Density Lipoprotein Cholesterol.

LDLC= Low DensityLipoprotein Cholesterol. Ox-LDLC=Oxidized LDLC

## Discussion

A significant and clinically relevant finding in this study was that the plasma level of Ox-LDLC was significantly increased in smokers and the level of significance was associated with increasing number of cigarettes and tobacco usage per day. Furthermore, this study showed a low level of mean total testosterone in smokers although this increase was not statistically significant, but was inversely correlated with Ox-LDLC. Thus suggesting that as the Ox-LDLC increases plasma T decreases. The possible reason for this is that after receptor -mediated endocytosis of LDLC, cholesterol removed from LDL is used for diverse metabolic processes required for healthy functioning cells including the synthesis of testosterone. However, modified/oxidized LDL is not recognised by the receptor and instead is taken up by scavenger receptor macrophages. The uptake of Ox-LDLC does not lead to its degradation. Instead it accumulates in the macrophages as cholesteryl esters, therefore foam cells and these amass in the sub-endothelial space, contributing to atherosclerotic plaques [18]. We could not demonstrate whether the low mean plasma testosterone proceeds or were a direct effect of smoking. More pronounced responses regarding hormones are found in most cases with heavy smokers. Since most of the subjects are light smokers, this could be the cause of the variance found between this study and those of others [19–21].

An epidemiology study in the North Eastern Nigeria [22] indicated that smoking is common among the low income earners. This was also revealed from the questionnaire on socioeconomic status used in this study. The reason for this is not known, but we speculate that since the majority of our subjects are motor park vendors/military men who had little or no education, and therefore do not have any idea on the pathophysiology of smoking. The possible mechanism linking low testosterone and elevated Ox-LDLC in smokers is plausible. Smoking is known to be a modifier of the male reproductive hormones, T [23, 24]. Its association with impaired lipid profile could be a major factor that promotes diseases like atherosclerosis and CVD especially in heavy smokers. Ox-LDLC is increasingly formed in smokers possibly, as a result of the reaction between the lipid fractions in LDL particles and the reactive oxygen species generated by the toxicants in cigarette and tobacco smoke [9, 25]. Cigarette and tobacco smoke are established source of free radicals generation [2] which increases oxidative stress and other associated complications like endothelium damage and lipid oxidation. A high level of Ox-LDLC in the body therefore, is deleterious. According to previous studies [26, 27], Ox-LDLC is also a major risk factor associated with atherosclerotic plaque formation. Significant and higher levels of plasma total cholesterol, triglyceride, and LDLC were obtained in this study. Several suggestions have been put forward; among which are that the effect of nicotine in the cigarette and tobacco smoke causes lipolysis through stimulation of catecholamine, and therefore increasing cholesterol, TG, free fatty acids and LDLC [20, 2]. Changes in lipid profile in smokers and the risk of increased tobacco related diseases have also been indicated to be dose dependent [28] and may as well be related to the duration of the habit. The role of dietary differences between smokers and non-smokers as well as free radical-mediated oxidative stress as a pivotal step for the development of atherosclerosis could also be relevant.

This study also demonstrated that smokers have significantly lower levels of plasma HDLC than the controls subjects. The reason for this low level is not clear; it may possibly be a pointer to a significant cardiovascular risk. Available evidence indicates that for every milligram decrease in plasma HDLC due to cigarette smoking or any other factors, there is a 2.5% chance of increased premature CVD development [29].

## Conclusion

The evidence from this study suggests a strong relationship between increased plasma Ox-LDLC and number of tobacco/cigarettes usage per day. The inverse correlation between testosterone and Ox-LDLC obtained in this study indicates that increases in Ox-LDLC can lead to reduce plasma testosterone in smokers. These changes in part, could lead to decreased fertility and premature CVD events in young adults.
